# Reach and uptake of mass drug administration for worm infections through health facility-, school-, and community-based approaches in two districts of Zambia: a call for scale-up

**DOI:** 10.1017/S0950268823000912

**Published:** 2023-06-08

**Authors:** Hikabasa Halwiindi, Lubombo Chooka, Masauso Moses Phiri, Buumba Tapisha, Sepiso K. Masenga, Jolezya Mudenda, Kingford Chimfwembe, Mwitwa Mugode, Benson M. Hamooya

**Affiliations:** 1 School of Public Health, University of Zambia, Lusaka, Zambia; 2 School of Medicine, Department of Pathology and Microbiology, University of Zambia, Lusaka, Zambia; 3 School of Medicine and Health Sciences, Mulungushi University, Livingstone, Zambia; 4 Chreso University, Faculty of Health Sciences, City Campus, Lusaka, Zambia

**Keywords:** Community-directed treatment, MDA, preschool-aged, school-aged, schistosomiasis, soil-transmitted helminths

## Abstract

Helminthiases cause significant health deficiencies among children. Mass administration of anthelminthic drugs has had significant results to counter these effects. We assessed the effects on and determinants of treatment coverage of community-directed treatment among children in Zambia, using cross-sectional survey data, and using chi-square test and multilevel mixed-effects model. Of 1,416 children, 51.5% were males and 48.5% were females, while 52.7%, were school-age, and 47.3% were preschool-age. Overall treatment coverage was 53.7% (95% confidence interval (CI) 51.1, 56.4). More preschool-age children were treated compared to school-age ones, 65.2% versus 43.4%, P < 0.001. Similarly, more children under community-directed intervention were treated compared to regular mass drug administration (65.2% versus 51.1 %, P < 0.001). Treatment among school-age participants was associated with being male (Adjusted Odds Ratio (AOR 1.83, 95%CI 1.23–2.72), receiving community-directed treatment (AOR 5.53; 95%CI 3.41–8.97), and shorter distance to health facility (AOR 2.20; 95%CI 1.36–3.56). Among preschool-aged participants, treatment was associated with being residents of Siavonga district (AOR 0.03; 95%CI 0.01–0.04) and shorter distance to health facility (AOR 0.35; 95%CI 0.21–0.59). Community-directed treatment can be used to increase treatment coverage, thereby contribute to 2030 vision of ending epidemics of neglected tropical diseases.

## Introduction

Soil-transmitted helminthiases (STH) and schistosomiasis (SCH) are neglected communicable parasitic infections that are endemic and constitute a considerable health problem in Zambia. Prevalences of STH and SCH of up to 42% have been reported in Zambia [1, 2]. The known conditions that accompany these infections include reduced food intake, interference with digestion and absorption of food, diminished nutritional status, reduced iron levels, iron-deficiency anaemia, vitamin A deficiency, and reduced physical and cognitive performance [3, 4]. Schistosome and STH infections have also been associated with increased transmission of HIV and the progression of AIDS to death through various mechanisms [5, 6].

There is ample evidence showing that the regular treatment of soil-transmitted helminthiases and schistosomiasis with deworming drugs produces immediate and long-term benefits that contribute to the health of children [7, 8]. Morbidity control of these infections can be achieved with inexpensive and highly effective drugs. There is enough evidence suggesting that Mass Drug Administration (MDA) initiatives for children are low-cost interventions and at the same time produce substantial benefits [9, 10]. These economies of scale are observed in both school-age and preschool children [8].

To avert the possible health and developmental consequences of these infections, the Ministry of Health introduced deworming programmes targeting all school-age children and preschool children (aged 12 to 59 months). Preschool children received the anthelminthic drug Mebendazole in a single dose twice a year during the child health week through health facilities. The school-age children received Mebendazole and Praziquantel through schools once a year. However, the proportion of children treated for soil-transmitted helminth and Schistosomes infections through the health-facility-based and school-based approaches is consistently low in certain areas of the country (unpublished data); this means that many children will not be treated regularly.

Community interventions that use their own members as drug distributors, such as the community-directed treatment (ComDT), and that aim to reduce morbidity caused by helminths have turned out to be important approaches for increasing treatment coverage [8, 11–13]. In this approach, the affected communities plan and implement the treatment programme after they have received the necessary information and training [14].

The United Nations’ third Sustainable Development Goal, which pledges to leave no one behind for universal health coverage, highlights the importance of ensuring no disparities in health outcomes and healthcare delivery. Neglected Tropical Diseases (NTDs) affect the most underprivileged populations and are therefore described as a litmus test for universal health coverage [15]. This means that in order to reach the 2030 neglected tropical disease elimination roadmap targets of eliminating soil-transmitted helminthiases and schistosomiasis as public health problems (defined as <1–2% of moderate to heavy intensity of infections), we need to ensure that the treatment reaches all children that require it [16].

A community-directed treatment project was implemented in two districts of Zambia in 2016 to assess the implementation, effectiveness, and sustainability of ComDT for soil-transmitted helminthiasis and schistosomiasis in preschool children and school-age children. This paper reports the treatment coverage and associated factors from a cross-sectional survey conducted after the second rounds of treatment in both preschool and school-age children. The findings of this survey are important because they help identify which children are ‘being left behind’ with our Mass Drug Administration (MDA) interventions, and where we need to scale up.

## Method

### Study area

The study was conducted in April and May of 2016 in the Mazabuka and Siavonga districts, in the southern province of Zambia. These districts were selected purposively based on the reported endemicity of soil-transmitted helminthiasis and schistosomiasis, and experience in implementation of ComDT (one with prior implementation of ComDT, Mazabuka; and the other one with no prior implementation of ComDT, Siavonga). The catchment population of the health center in each area formed the cluster, the local geographical area. The local geographical areas for the two districts were Magoye (16.0017S, 27.6041E) and Munjile (16.019 2S, 27.5010E) in Mazabuka district, and Matuwa (16.4004S, 28.6843E) and Nabutezi (16.4339S, 28.5433E) in Siavonga district. Nabutezi’s local geographical area comprised catchment populations for two health centers (Nabutezi and Manchamvwa health centers); this was because of the small sampling frames at Nabutezi health center. In each district, one local geographical area was randomly allocated to the intervention strategy (ComDT). Geographically, Siavonga district lies 200 km from the capital city Lusaka, and is very mountainous and spreads out along the north bank of Lake Kariba in the Zambezi Valley. Fishing is a major activity and source of livelihood for local villagers. Mazabuka district lies 120 km south of the capital city Lusaka, and the terrain is generally flat with numerous small rivers. The majority of the population in the Magoye and Munjile local geographical areas are subsistence farmers involved in cash crop production. Additional details on the study areas are published elsewhere [17, 18].

### Summary of main study

The current study was part of a cluster-randomised project that was investigating the implementation, scale-up, and sustainability of integrated ComDT in school-age children and preschool children. Using the ComDT approach, the villagers were fully in charge of selecting, organising, and implementing the distribution of drugs to children in their villages. The selected community drug distributors (CDDs) were trained and provided with free anthelminthic drugs (Mebendazole and Praziquantel) to treat the children every six months. Only those children who missed out on receiving treatment from the health facilities and schools were treated by the community drug distributors. The preschool children were given Mebendazole and vitamin A, whereas the school-age children received Mebendazole and Praziquantel. The monitoring of the intervention process documented the process of implementation of ComDT, to address in particular the following: i) the selection of distributors in the community-directed treatment arm; ii) the operationalisation of treatment; iii) potential sustainability; and iv) the factors affecting scaling up.

### Study design

The current study is a cross-sectional survey that was part of a cluster-randomised study. The cluster-randomised study had the following combinations of interventions:

1. ComDT clusters (intervention) = [ComDT for preschool children and school-age children] + [Health-Facility approach for preschool + school-based approach for school-age children]
2. Routine Mass Drug Administration (rMDA) clusters: [Health-Facility approach for preschool children + school-based approach for school-age children] as they usually run.

The health-facility approach means that the preschool children received treatment at a health facility. The school-based approach means that the school-age children received treatment at school.

### Sample size determination

The sample size for this cross-sectional study was based on the sample size determined for the cluster-randomised study, and used the following outcome variable: proportion of children treated. Using the projected change in treatment coverage between the baseline and end line, the sample size for each cluster was determined as follows:
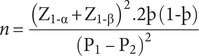

Where: P = the percentage frequency of outcome (i.e. treatment coverage) in each group

þ = the mean of proportions in both groups, 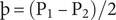



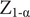
 = significance level (0.05 i.e. Z = 1.96)


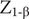
 = the power level (90% i.e. Z = 1.28)

The World Health Assembly (WHA52.19) recommended regular treatment coverage of 75% for effective control of schistosomiasis and soil-transmitted helminthiases [19]. The average deworming treatment coverage for different interventions in Zambia was 80% (i.e. 106% of the target) [20]. We estimated that the difference in treatment coverage between the routine treatment approaches and the community-directed treatment approach would be 20% based on a systematic review [11]. Therefore, estimating the percentage of children treated in the study period under the health-facility- and school-based approaches to be 60%, respectively, and that in the ComDT approach in addition to the health-facility approach and to the school-based approach to be 80%, the required sample size for surveys in each cluster (health center) was 110. To account for the design effect (Design Effect = 2) from cluster sampling, the required sample size was doubled to 220. To account for the loss to follow-up and withdrawals from the study, the number of participants was increased by 30%, giving a sample size of 286 per local geographical area. A minimum total of 1,144 children was required in both districts, that is, 572 preschool and school-age children, respectively. A total of 1,416 children were sampled for this survey.

### Eligibility criteria

The study included preschool children aged 12 to 59 months and considered as residents in the local area. The eligibility criterion for school-age children was being aged between 5 and 19 years old and considered as a resident in the local area. A census was conducted in the selected local geographical areas in both districts to determine the sampling frame. An eligible child who was away during the census but was likely to be back home during the next child health week and school treatment exercise was included in the census. Children with unconfirmed dates of birth, who were non-residents of the area, who would not be around during the next treatment cycle, or who had taken Mebendazole (or albendazole) and Praziquantel just before the treatment during child health week and school-based treatment were excluded.

### Outcome measures

The main outcome measure was treatment coverage, that is, the percentage of children that received Mebendazole and/or Praziquantel. Treatment was through the school-based, health-facility-based, or community-based MDA.

### Data collection

The data for this paper were collected during a household survey conducted to determine which children were treated through the health-facility arm, the school-based arm, and the ComDT approach. This was done after the second round of treatment of the main trial. At baseline, we recruited 10 data collectors through the village headmen. The data collectors were trained for two days on field techniques on how to conduct a census, locate participants, collect urine and feacal samples, and conduct interviews. The training involved classroom sessions, role-play, and piloting in the field. Demographic information was collected from each household in the study villages during the census. A household was defined as ‘a family structure whose members shared the same kitchen and whose members were regarded as being under the care of one particular household head’ [17]. A unique identity number (ID) was given to each eligible child during the census.

To determine treatment coverage, 1,416 households were selected from the sampling frames for preschool and school-age children; 572 for each age group. Only one child was selected from the sampled households. Where a household randomly selected had both a preschool and a school-age child, the preschool child was selected, and the next-door household was selected for the recruitment of the school-age child. Verbal consent was obtained from the caregivers of the children, and assent was further sought from the school-age children before proceeding with the interview. The treatment status of each child was determined in two steps: by recall and by confirmation with treatment record. The IDs of the children who were treated under each cluster and intervention type were recorded in treatment registers. For preschool children, the caregivers were asked whether the child was treated during the last child health week, and, if so, by whom. The treatment status was confirmed by requesting for an under-five card that has a record of treatment. Similarly, school-age children were first asked whether they received treatment. Treatment status was then confirmed by reviewing both school and community treatment records. The sequence of events was as follows: –routine MDA followed by ComDT to cater to non-school-going school-aged children and preschool children who were not treated at the health facility after which a census was done before the household survey.

### Data analysis

All statistical analyses were done using Stata 15 (StataCorp, College Station, Texas, USA). Descriptive statistics were used to characterise the distribution of socio-demographic variables among the study participants. The chi-square test was used to determine relationships between two categorical variables. A two-sample test of proportions was used to ascertain the statistical difference between two proportions arising from independent samples. The multivariable multilevel mixed-effects model was used to determine the factors associated with schistosomiasis treatment among school-age and preschool participants. The variables in the final model were selected based on the previous literature and significance at bivariate analysis. For statistical significance, 95% confidence interval (CI) and p-value of less than 5% were used. Missing values were not included in the analysis.

## Results

### Profile of participants

The total number of sampled children was 1,416, out of which 51.5% (*n* = 695) were males, 47.3 percent (*n* = 670) were preschool children, and 52.7% (*n* = 746) were school-age children. A slightly higher proportion of school-age and preschool participants were from Siavonga (53.4%, *n* = 398) and Mazabuka districts (59.4%, *n* = 398), respectively. A higher proportion of the children were not going to school (55.6%, *n* = 727) compared with those who were going (44.4%, *n* = 581). For school-age children, a higher proportion was enrolled in school (76.7%, *n* = 561) compared to those that were out of school (23.3%, *n* = 170). 58.4 percent (*n* = 827) of the study participants were from the ComDT intervention clusters and 42% (*n* = 589) from the rMDA clusters. See Table 1.

**Table 1.** Composition of study population by age group

			Stratified by age group, *n* (%)	
Variable		Aggregated, *n* (%)	School age	Preschool
Total population		*N* = 1,416	746 (52.7)	670 (47.3)
District	Mazabuka	746 (52.7)	348 (46.7)	398 (59.4)
	Siavonga	670 (47.3)	398 (53.4)	272 (40.6)
Local geographical area	Nabutezi (Siavonga)	381 (26.9)	264 (35.4)	117 (17.5)
	Matuwa (Siavonga)	288 (20.3)	133 (17.8)	155 (23.1)
	Magoye (Mazabuka)	301 (21.3)	124 (16.6)	177 (26.4)
	Munjile (Mazabuka)	446 (31.5)	225 (30.2)	221 (33.0)
Sex^a^	Female	654 (48.5)	341 (47.6)	313 (49.5)
	Male	695 (51.5)	376 (52.4)	319 (50.5)
Child goes to school^a^	No	727 (55.6)	170 (23.3)	557 (96.5)
	Yes	581 (44.4)	561 (76.7)	20 (3.5)
Treatment approach	rMDA	589 (41.6)	257 (34.5)	332 (49.6)
	iComDT intervention	827 (58.4)	489 (65.6)	338 (50.5)
Distance to health facility far^a^	No	552 (39.2)	303 (40.7)	249 (37.4)
	Yes	858 (60.9)	442 (59.3)	416 (62.6)
Medication safe^a^	No	7 (0.5)	3 (0.4)	4 (0.6)
	Yes	1399 (99.5)	735 (99.6)	664 (99.4)
Worms are important health matter	No	290 (20.7)	140 (19.1)	150 (22.5)
	Yes	1109 (79.3)	592 (80.9)	517 (77.5)

iComDT, integrated community-directed treatment; rMDA, routine Mass Drug Administration.

a Variables had missing values of <10%.

### Chi-square test of the relationship between treatment and age group (school-age and preschool)

Table 2 shows the relationship between treatment and age group (school-age and preschool) among children of Mazabuka and Siavonga. Overall treatment coverage was 53.7% (*n* = 761, 95% confidence interval (CI) 51.1, 56.4). A significantly higher proportion of preschool-age children were treated compared with school-age ones, 65.2% versus 43.4%, *P* < 0.001.

**Table 2.** Demographic factors and treatment intervention sorted according to treatment status

		School-age participants, n = 746					
		Treated, *n* (%)	Treated, *n* (%)		Preschool participants, n = 670		
Variable		Yes, 324 (43.4)	No, 422 (56.6)	*P*-value	Yes, 437 (65.2)	No, 233 (34.8)	*P*-value
Total population							
District	Mazabuka	256 (79.0)	92 (21.8)	**<0.001**	370 (84.7)	28 (12.0)	**<0.001**
	Siavonga	68 (21.0)	330 (78.2)		67 (15.3)	205 (88.0)	
Local geographical area	Nabutezi (Siavonga)	41 (12.6)	223 (52.8)	**<0.001**	26 (6.0)	91 (39.1)	**<0.001**
	Matuwa (Siavonga)	26 (8.0)	107 (25.4)		41 (9.4)	114 (48.9)	
	Magoye (Mazabuka)	48 (14.8)	76 (18.0)		164 (37.5)	13 (5.6)	
	Munjile (Mazabuka)	209 (64.5)	16 (3.8)		206 (47.1)	15 (6.4)	
Sex^a^	Female	129 (43.1)	212 (50.7)	**0.045**	202 (50.3)	111 (48.3)	0.631
	Male	170 (56.9)	206 (49.3)		200 (49.7)	119 (51.7)	
Child goes to school^a^	No	54 (16.9)	116 (28.2)	**<0.001**	359 (97.3)	198 (95.2)	0.186
	Yes	265 (83.1)	296 (71.8)		10 (2.7)	10 (4.8)	
Treatment approach	rMDA	74 (22.8)	183 (43.4)	**<0.001**	205 (46.9)	127 (54.5)	0.061
	iComDT intervention	250 (77.2)	239 (56.6)		232 (53.1)	106 (45.5)	
Distance to health facility far^a^	No	136 (42.0)	167 (39.7)	0.525	201 (46.4)	48 (20.7)	**<0.001**
	Yes	188 (58.0)	254 (60.3)		232 (53.6)	184 (79.3)	
Medication safe^a^	No	4 (1.2)	15 (3.6)	**0.046**	4 (0.9)	16 (6.9)	**<0.001**
	Yes	319 (98.8)	405 (96.4)		431 (99.1)	215 (93.1)	
Worms are important health matter	No	87 (26.9)	53 (13.0)	**<0.001**	118 (27.1)	32 (13.8)	**<0.001**
	Yes	237 (73.1)	355 (87.0)		317 (72.9)	200 (86.2)	

iComDT, integrated community-directed treatment; rMDA, routine Mass Drug Administration.

a Variables had missing values of <10%.

Bold *P*-values signify statistically significant results

#### School-age participants

A significantly higher proportion of children from Mazabuka were treated as compared with those from Siavonga, 79.0% (*n* = 256) versus 21.8% (*n* = 92), *P* < 0.001. Among school-age children, there was a higher proportion of males in the treated group than in the untreated group (56.9%, *n* = 170 versus 49.3%, *n* = 206, *P* = 0.045. Similarly, there was a higher proportion of school-going children in the treated group than in the untreated group, 83.1% versus 71.8%, *P* < 0.001. There was a higher proportion of school-age children from the ComDT intervention area in the treated group in comparison with the not-treated group (77.2%, *n* = 250 versus 56.6% 298, *n* = 239, *P* < 0.001). For participants whose caretakers said medication was safe, a higher percentage of the children were in the treated group as compared to those in the not-treated group (98.8% versus 96.4%, *P* = 0.046). A higher proportion of the children whose caretakers did not identify worms as important health matters were in the treated group as compared with those in the not-treated group, 26.9% versus 13.0%, *P* < 0.001.

#### Preschool participants

A greater percentage of preschool children from Mazabuka were in the treated group in comparison with those who were in the not-treated group, 84.7% versus 12.0%, *P* < 0.001. Among the children whose caretakers said that distance to the health facility was far, a lower proportion was in the treated group in comparison to the not-treated group (53.6% versus 79.3% versus *P* < 0.001). A higher percentage of the participants whose caretakers said medication is safe were in the treated group as compared to those in the not-treated, 99.1% versus 93.1%, *P* < 0.001. Similarly, a higher proportion of the children whose caretakers did not identify worms as an important health matter were in the treated group as compared to the not-treated group (27.1% versus 13.8%, *P* < 0.001).

### Bivariate and multivariable analysis of factors associated with treatment among school-age and preschool children

Table 3 shows the bivariate and multivariable analysis of factors associated with worm infection treatment for school-age and preschool children in the Mazabuka and Siavonga districts.

**Table 3.** Bivariate and multivariable analysis of factors associated with SCH and STH treatment among children of Mazabuka and Siavonga districts

	School-age participants						Preschool participants					
	Bivariate analysis			Multivariable analysis			Bivariate analysis			Multivariable analysis		
Characteristics	OR	95%CI	*P*-value	AOR	95%CI	*P*-value	OR	95%CI	*P*-value	AOR	95%CI	*P*-value
*District*												
Mazabuka	1			1			1			1		
Siavonga	0.29	0.01–6.62	0.435				0.02	0.01–0.04	**<0.001**	0.03	0.01–0.04	**<0.001**
*Local geographical area*												
Nabutezi (Siavonga)	1			1			1			1		
Matuwa (Siavonga)	1.32	0.77–2.28	0.313				1.26	0.71–2.21	0.424			
Magoye (Mazabuka)	3.44	2.10–5.62	**<0.001**				44.16	21.64–90.13	**<0.001**			
Munjile (Mazabuka)	71.06	38.70–130.50	**<0.001**				48.08	24.31–95.06	**<0.001**			
*Sex*												
Female	1			1			1			1		
Male	1.76	1.15–2.70	**0.009**	1.83	1.23–2.72	**0.003**	1.05	0.67–1.65	0.828	0.94	0.58–1.51	0.788
*Child goes to school*												
No	1			1								
Yes	1.03	0.64–1.66	0.908	0.85	0.52–1.41	0.533						
*Treatment approach*												
rMDA	1			1			1			1		
iComDT intervention	4.64	3.04–7.07	**<0.001**	5.53	3.41–8.97	**<0.001**	0.89	0.57–1.40	0.612	1.06	0.63–1.78	0.824
*Distance to health facility far*												
No	1			1			1			1		
Yes	1.27	0.81–2.00	0.293	2.20	1.36–3.56	**0.001**	0.39	0.24–0.65	**<0.001**	0.35	0.21–0.59	**<0.001**
*Medication safe*												
No	1			1			1			1		
Yes	1.84	0.46–7.34	0.389	0.41	0.08–2.02	0.276	3.37	0.80–14.30	0.099	3.73	0.89–15.67	0.072
*Worms are important health matter*												
No	1			1			1			1		
Yes	1.71	0.90–3.22	0.100	1.45	0.84–2.50	0.179	1.42	0.76–2.65	0.265	1.26	0.64–2.49	0.504

CI, confidence interval; OR, odds ratio; rMDA, routine mass drug administration.

iComDT integrated community-directed treatment, bold *P*-value <0.05.

*School-age participants*: In bivariate analysis, children from Magoye and Munjile were 3.44 and 71.06 times more likely to be treated as compared with children from Nabutezi, *P* < 0.001. Male children had 76% increased odds of being treated as compared with their female counterparts, *P* = 0.009. Children under the ComDT intervention were 4.64 times more likely to be treated as compared with those in the rMDA, *P* < 0.001. In multivariable analysis, male children had an 83% increased chance of being treated as compared with female children, *P* = 0.003. Participants in the ComDT intervention were 5.53 times more likely to be treated as compared to the participants in the rMDA, *P* < 0.001. Children whose parents felt that the distance to a health facility was high had 2.20 increased odds of being treated in comparison to those who felt that the health facility was not far, *P* = 0.001. Note that there was no association between going to school and treatment shown in bivariate and multivariable analysis (Table 3) when there was an association shown in the chi-square test results (Table 2). The discrepancy could result from accounting for clustering in Table 3, which was not done in Table 2. This could have attenuated the effect of the variable ‘going to school’ on treatment.

*Preschool participants*: In bivariate analysis, children from Siavonga had 98% reduced odds of being treated as compared with those from Mazabuka, *P* < 0.001. Children whose parents felt that the distance to a health facility was high had a 61% reduced chance of being treated in comparison to those who felt that the health facility was not far, *P* < 0.001. In multivariable analysis, children from Siavonga had 97% reduced odds of being treated as compared with those from Mazabuka, *P* < 0.001. Children whose parents felt that the distance to a health facility was high had a 65% reduced chance of being treated in comparison to those who felt that the health facility was not far, *P* < 0.001.

## Discussion

The current study aimed at determining which children were treated and which ones were missed out by the routine mass drug administration and the ComDT approaches. The study also investigated the factors associated with the treatment in children. Great progress has been made to control and eliminate NTDs through national public health programs [15]. However, people affected by NTDs are often missed by public health campaigns because of the barriers to NTD treatment that still exist [21], and that prevent equitable access to much-needed health services. To attain the third Sustainable Development Goal (SDG3) of universal health coverage, we need to know how much more we can do in our neglected tropical diseases programming in order not to leave anyone behind. And in order to ‘leave no one behind’, we need to know who we are missing out on with our current efforts.

On aggregate, a significantly higher proportion of children received treatment in the ComDT+rMDA areas compared to the routine mass drug administration areas. The ComDT+rMDA treatment approach was significantly associated with increased treatment coverage among school-age children, with five times higher chance of being treated when compared to the routine mass drug administration. The probable reason could be the mop-up effect of community drug distribution that augmented routine mass drug administration done in school for those who were left out due to absenteeism, non-enrollment, and other reasons. Besides, with the majority of the school-age participants attending school, most of them could have been treated under the rMDA. This is supported by earlier studies indicating that school-age children enrolled in school were more likely to be treated compared to out-of-school school-age children [21]. Furthermore, although not significantly associated, aggregated figures show that more preschool children were treated under the ComDT approach compared to the rMDA. The higher treatment coverage in the ComDT+rMDA means that the ComDT approach is still an important implementation strategy for raising the likelihood of treatment among children. These results validate previous studies [17, 22, 23] that found that ComDT interventions increased treatment coverage and outperformed rMDA in reaching the target populations. Some key attributes to achieve high reach and treatment coverage have been identified and include the training and motivation of community drug distributors, and the responsive and agile implementation strategy, engagement, and availability of the community [24]. Although the ComDT intervention increased treatment coverage significantly in the ComDT+rMDA clusters, there were still children in both the intervention and the control areas that still missed out on receiving treatment. This highlights the need to resolve the long-standing need of having special strategies to reach this group in order for them to benefit from treatment, and also for them not to be reservoirs of infections in their communities. There is a need to ensure ‘no one is left behind’ amongst the most marginalised groups, such as the out-of-school school-age children. The current study as well as earlier ones have identified medical, personal, and operational predictors of treatment coverage of ComDT interventions [25–27].

Gender is one of the important determinants of treatment among school-age children, with males more likely to be treated than females. Several factors could be responsible for this, such as the traditional beliefs of educating boys as compared to girls, and also the relatively higher drop-out levels of girls as compared to boys over the years of primary and secondary school, leading to numerical under-representation [28]. Furthermore, studies have shown that gender-based stereotypes lead to disparities in accessing education and healthcare services [29]. Other factors that may have contributed to the reduced odds of females being treated may be their absentia from school during the treatment period due to menstrual issues and also the higher chances of them helping out with household chores where males are exempted.

Distance to the facility was one of the factors associated with treatment among preschool children, with longer distances indicating a reduction in the likelihood of treatment. With most rural households scattered far away from each other, the placement of health centers or health posts is a challenge, and hence an area deemed central to all the households is chosen even though households may be far. This means caregivers have to carry their children over long distances. Besides, in households where there are many preschool children, only those who can be carried receive treatment, leaving others untreated. This acts as a deterrent to the uptake of health services, such as those offered during child health week [18]. Several other studies have also found that distance has a negative effect on health service uptake, including services tailored or related to child care [30, 31]. The situation is further compounded by a lack of transport for health workers, mothers, and community health workers [18]. Another probable reason could be that the caregivers may not take a non-symptomatic illness that does not threaten the child’s life seriously. Addressing this challenge requires investment in transportation for community health facilitators and health workers as well as strengthening outreach services. On the other hand, school-age children were more likely to be treated despite the long distance to the school. This probably is due to the fact that they received treatment at school. It should also be noted that earlier studies found that school-age children enrolled in school were more likely to be treated compared to those out of school as the focus of rMDA was purely within schools [21].

The variable ‘district’ was one of the strong predictors of treatment. A child in Mazabuka district was more likely to be treated compared to one in Siavonga. One possible reason was that the clusters in Mazabuka district have had prior experience in implementing the ComDT intervention for soil-transmitted helminth infections from 2006 to 2010, whereas it was the first time that clusters in Siavonga district were using the ComDT approach. This means Mazabuka district had more experience, thereby making community engagement and participation easier; confidence in the community drug distributors higher, and acceptability of the ComDT strategy higher. This supports earlier findings that suggest that community participation and ownership of strategies like the ComDT may take some time to develop within communities [32]. This calls our attention to the need for strong community engagement and ownership of community-based interventions like ComDT, especially in new sites, if the full benefit of these interventions is to be accrued to the target population groups.

Low levels of community participation and ownership of the ComDT intervention have been seen to result in low treatment coverage because of poor ownership of the process: that is, from the selection of community drug distributors, to the selection of the time when drug distribution takes place, among other processes [32]. In addition to intervention-related factors, the difference in terrain in the two districts could have contributed to the difference in treatment coverage. Mazabuka district is flat land, whilst Siavonga is very mountainous. This means that travel to treatment sites under the rMDA for children and caregivers was more challenging in Siavonga district. In addition, in ComDT clusters, travel by community drug distributors as well as by caretakers with children was also a challenge in Siavonga district; thereby fewer children reached for treatment. Large catchment areas coupled with long travel distances have been identified as common challenges that community drug distributors face under the ComDT interventions [18]. Provision of transportation like bicycles, and reducing the catchment area covered by each community drug distributor, coupled with increased numbers of community drug distributors have been identified as strategies to mitigate this challenge.

Some of the limitations of the study could be the inclusion of school-aged children who may not have taken the medication but found themselves in the school treatment register by virtue of their presence at school. Furthermore, those who could not recall the MDA event taking place at their school could have re-registered as having been not treated, hence taking getting treated a second time under the community-directed treatment. Confirmation of treatment in the under-five card in preschool children eliminated the recall bias that could otherwise compromise the data.

## Conclusion

Identifying who we are leaving behind is the first step in closing the gap to achieving the 2030 elimination goals for schistosomiasis and other neglected tropical diseases, by scaling up interventions that increase the uptake of preventive chemotherapy coverage in the at-risk subgroups. A shift in intervention approaches is therefore needed. Participatory approaches like ComDT that engage communities, ensuring their interests and realities are heard and taken into account, offer hope of reaching the marginalised groups. Implementation research is needed that will focus on sustaining the gains made by these participatory methods and help to develop and adapt strategies to reach the marginalised population groups.

## Data Availability

The data on which the findings reported in this study are based have been presented in the article. Additional data for this study are available from the corresponding author upon request.
